# Poly[[(μ_3_-adamantane-1,3-di­carboxyl­ato)aqua­[μ-*N*-(pyridin-3-yl)isonicotinamide]­nickel(II)] monohydrate], a layered coordination polymer with (4,4) topology

**DOI:** 10.1107/S2414314623007587

**Published:** 2023-09-08

**Authors:** Jamelah Z. Travis, Robert L. LaDuca

**Affiliations:** a Department of Chemistry, Hope College, Holland, MI 49423, USA; b E-35 Holmes Hall, Michigan State University, Lyman Briggs College, 919 E. Shaw Lane, East Lansing, MI 48825, USA; Katholieke Universiteit Leuven, Belgium

**Keywords:** crystal structure, coordination polymer, adamantane-1,3-di­carboxyl­ate, *N*-(pyridin-3-yl)isonicotinamide, nickel(II)

## Abstract

The title com­pound, {[Ni(C_12_H_14_O_4_)(C_11_H_9_N_3_O)(H_2_O)]·H_2_O}_*n*_, contains octa­hedrally coordinated Ni^II^ ions ligated by adamantane-1,3-di­carboxyl­ate (adc) and *N*-(pyridin-3-yl)isonicotinamide (3-pina) ligands forming coordination polymer layers with a (4,4) grid topology. These diperiodic layer motifs aggregate in an *AAA* pattern mediated by supra­molecular C—H⋯O inter­actions to form the full triperiodic crystal structure of the title com­pound.

## Abstract 2

The title com­pound contains octa­hedrally coordinated Ni^II^ ions ligated by adamantane-1,3-di­carboxyl­ate and *N*-(pyridin-3-yl)isonicotinamide ligands forming coordination polymer layers with a (4,4) grid topology.

## Graphical abstract

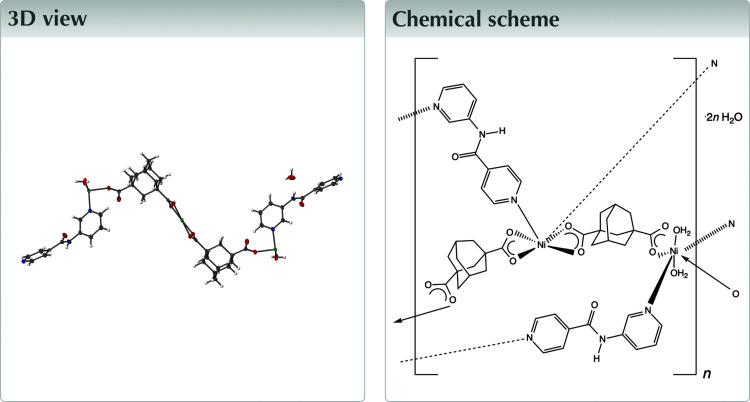

## Structure description

The title com­plex was obtained during attempts to prepare divalent nickel coordination polymers featuring adamantane-1,3-di­carboxyl­ate (adc) ligands and hydro­gen-bonding-capable di­pyridyl­amide ligands. We have reported nickel adc coordination polymers featuring 4,4′-di­pyridyl­amine (dpa) (Travis *et al.*, 2018 ▸). {[Ni(adc)(dpa)]·6.5H_2_O}_*n*_ manifests a stacked arrangement of (4,4) rectangular-grid diperiodic coordination polymer motifs, while the crystal structure of the partially protonated compound {[Ni_2_(adc)(adcH)_2_(dpa)_2_]·H_2_O}_*n*_ displays an uncommon 10^3^ topology triperiodic **srs** network.

**Figure 1:**
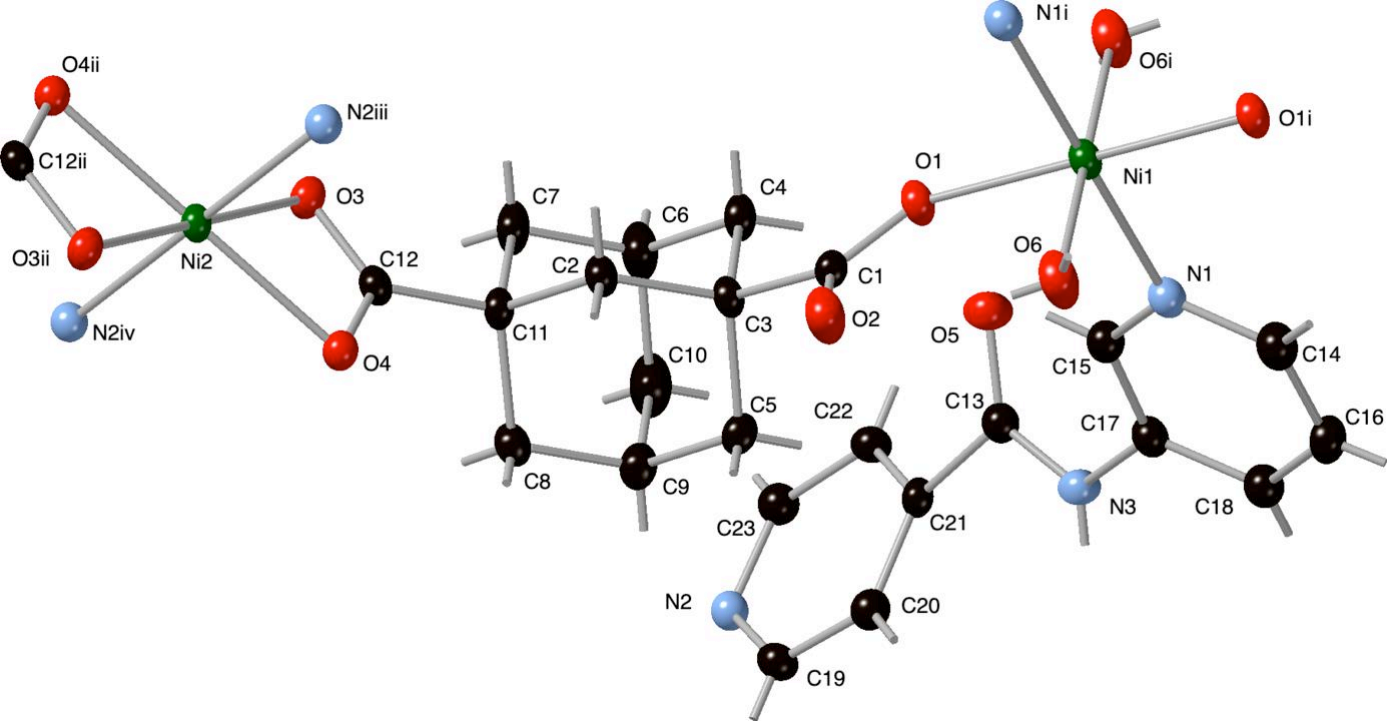
The nickel coordination environments in the title com­pound with a full ligand set. Displacement ellipsoids are drawn at the 50% probability level. Color code: Ni green, O red, N light blue, and C black. H-atom positions are shown as gray sticks. Symmetry codes are as listed in Table 1 ▸.

The asymmetric unit of the title com­pound, {[Ni(adc)(3-pina)(H_2_O)]·H_2_O}_*n*_, contains two Ni^II^ atoms on crystallographic inversion centers (Ni1 and Ni2), a com­plete adc ligand, an *N*-(pyridin-3-yl)isonicotinamide (3-pina) ligand, one water mol­ecule bound to Ni1, and one water mol­ecule of crystallization (Fig. 1 ▸). Operation of the crystallographic inversion centers generates two distinct coordination environments. The Ni1 atoms possess an octa­hedral {N_2_O_4_} coordination environment with *trans* aqua ligands, *trans* O-atom donors from two adc ligands, and *trans* 3-pyridyl N-donor atoms from two 3-pina ligands. The Ni2 atoms also display an octa­hedral {N_2_O_4_} coordination environment, but there are no bound water mol­ecules. Instead, 4-pyridyl N-donor atoms belonging to the isonicotinamide termini of two 3-pina ligands adopt the nominal *trans*-axial positions. The nominal equatorial plane at the Ni2 atoms are taken up by chelating carboxyl­ate groups belonging to two adc ligands. The bond lengths and angles within the coordination environment are listed in Table 1 ▸.

**Figure 2:**
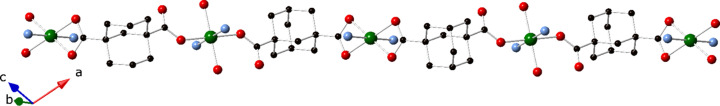
The [Ni_2_(H_2_O)_2_(adc)_2_]_*n*_ coordination polymer chain motif in the title com­pound.

**Figure 3:**
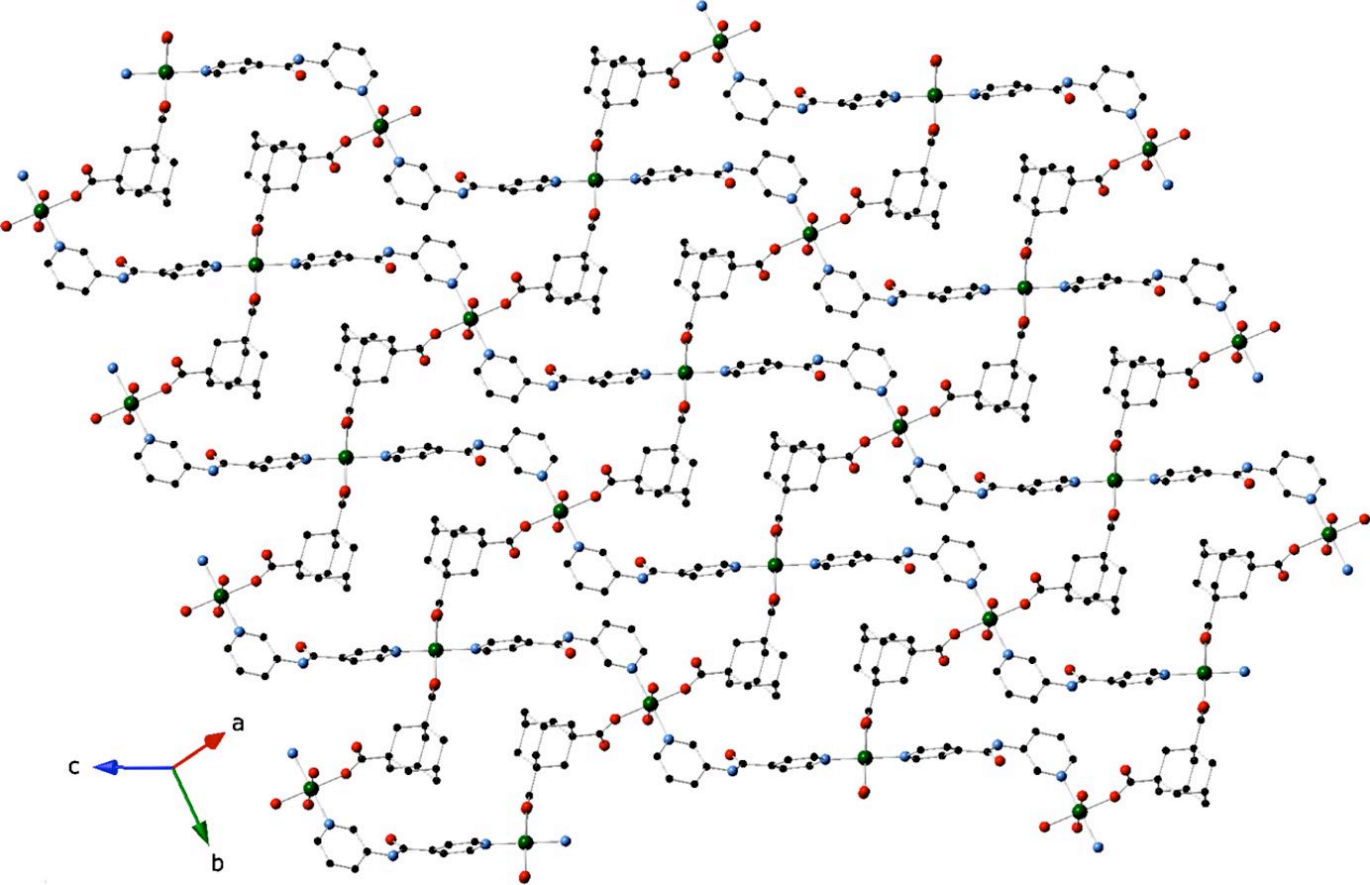
The [Ni_2_(H_2_O)_2_(adc)_2_(3-pina)_2_]_*n*_ coordination polymer layer motif in the title com­pound.

**Figure 4:**
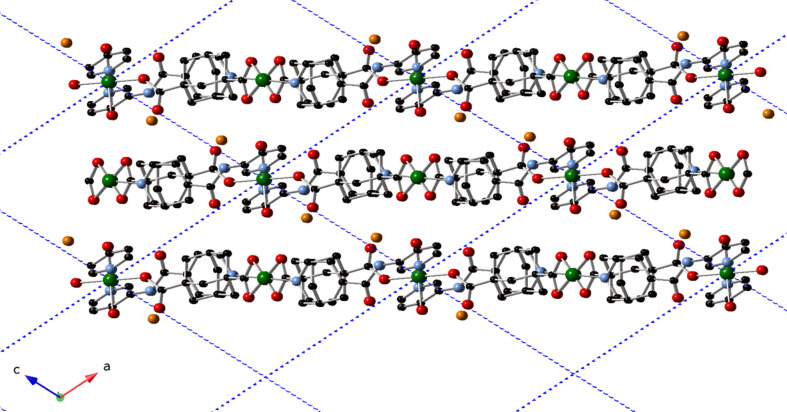
The *AAA* stacking of coordination polymer layers in the title com­pound.

The adc ligands adopt a chelating/monodentate binding mode, producing [Ni_2_(H_2_O)_2_(adc)_2_]_*n*_ monoperiodic chain motifs with an Ni1⋯Ni2 through-ligand distance of 9.694 (1) Å (Fig. 2 ▸). These are arranged parallel to the [1 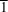 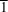 ] direction. In turn, the chain motifs are linked into diperiodic [Ni_2_(H_2_O)_2_(adc)_2_(3-pina)_2_]_*n*_ coordination polymer layer motifs with (4,4) grid topology (Fig. 3 ▸); these are oriented parallel to the (101) crystal planes. The bound water mol­ecules (O6) engage in hydro­gen bonding to adc carboxyl­ate O atoms (O2 and O3) (Table 2 ▸). Water mol­ecules of crystallization are held to the layer motifs by N—H⋯O hydro­gen-bonding inter­actions involving the 3-pina amide groups (Table 2 ▸). Nonclassical C—H⋯O hydro­gen-bonding inter­actions [C22⋯O5 distance = 3.110 (1) Å] promote aggregation of the [Ni_2_(H_2_O)_2_(adc)_2_(3-pina)_2_]_*n*_ layers into the triperiodic full crystal structure of the title com­pound. The layers stack in an *AAA* pattern along both the *a* and the *c* crystal directions (Fig. 4 ▸).

## Synthesis and crystallization

Ni(NO_3_)_2_·6H_2_O (108 mg, 0.37 mmol), adamantane-1,3-di­car­b­oxy­lic acid (adcH_2_) (93 mg, 0.37 mmol), *N*-(pyridin-3-yl)iso­nicotinamide (3-pina) (74 mg, 0.37 mmol), and 0.75 ml of a 1.0 *M* NaOH solution were placed in 10 ml of distilled water in a Teflon-lined acid digestion bomb. The bomb was sealed and heated in an oven at 393 K for 48 h, and then cooled slowly to 273 K. Green crystals of the title com­plex were obtained in 71% yield.

## Refinement

Crystal data, data collection and structure refinement details are summarized in Table 3 ▸. H atoms attached to O atoms were located in a difference Fourier map and refined freely with *U*_iso_(H) values fixed at 1.5*U*_eq_(O).

## Supplementary Material

Crystal structure: contains datablock(s) I, 1R. DOI: 10.1107/S2414314623007587/vm4062sup1.cif

Structure factors: contains datablock(s) I. DOI: 10.1107/S2414314623007587/vm4062Isup2.hkl

CCDC reference: 2291890

Additional supporting information:  crystallographic information; 3D view; checkCIF report

## Figures and Tables

**Table 1:** Selected geometric parameters (Å, °)

Ni1—O1^i^	2.0216 (19)	Ni2—O3	2.0766 (18)
Ni1—O1	2.0216 (19)	Ni2—O3^ii^	2.0766 (18)
Ni1—O6^i^	2.079 (2)	Ni2—O4	2.1113 (18)
Ni1—O6	2.079 (2)	Ni2—O4^ii^	2.1113 (18)
Ni1—N1	2.138 (2)	Ni2—N2^iii^	2.058 (2)
Ni1—N1^i^	2.138 (2)	Ni2—N2^iv^	2.058 (2)
			
O1^i^—Ni1—O1	180.0	O3^ii^—Ni2—O3	180.0
O1—Ni1—O6^i^	90.58 (8)	O3—Ni2—O4^ii^	116.95 (7)
O1—Ni1—O6	89.42 (8)	O3—Ni2—O4	63.05 (7)
O1^i^—Ni1—O6^i^	89.42 (8)	O3^ii^—Ni2—O4	116.95 (7)
O1^i^—Ni1—O6	90.58 (8)	O3^ii^—Ni2—O4^ii^	63.05 (7)
O1—Ni1—N1	90.06 (8)	O4—Ni2—O4^ii^	180.0
O1—Ni1—N1^i^	89.94 (8)	N2^iii^—Ni2—O3^ii^	92.34 (8)
O1^i^—Ni1—N1^i^	90.06 (8)	N2^iii^—Ni2—O3	87.67 (8)
O1^i^—Ni1—N1	89.94 (8)	N2^iv^—Ni2—O3	92.33 (8)
O6^i^—Ni1—O6	180.0	N2^iv^—Ni2—O3^ii^	87.66 (8)
O6—Ni1—N1	93.49 (9)	N2^iv^—Ni2—O4	94.86 (8)
O6^i^—Ni1—N1	86.51 (9)	N2^iii^—Ni2—O4	85.14 (8)
O6^i^—Ni1—N1^i^	93.49 (9)	N2^iii^—Ni2—O4^ii^	94.86 (8)
O6—Ni1—N1^i^	86.51 (9)	N2^iv^—Ni2—O4^ii^	85.14 (8)
N1—Ni1—N1^i^	180.0	N2^iii^—Ni2—N2^iv^	180.0

Symmetry codes: (i) 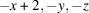 ; (ii) 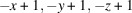 ; (iii) 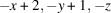 ; (iv) 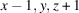 .

**Table 2:** Hydrogen-bond geometry (Å, °)

*D*—H⋯*A*	*D*—H	H⋯*A*	*D*⋯*A*	*D*—H⋯*A*
O6—H6*A*⋯O3^v^	0.87 (1)	2.04 (2)	2.886 (3)	163 (3)
O6—H6*B*⋯O2^i^	0.85 (2)	1.86 (2)	2.684 (3)	163 (3)
N3—H3⋯O1*W*	0.88	1.94	2.769 (3)	157
O1*W*—H1*WA*⋯O4^vi^	0.84 (2)	2.00 (2)	2.831 (3)	169 (4)
O1*W*—H1*WB*⋯O2^iii^	0.84 (2)	2.10 (2)	2.921 (3)	165 (4)

Symmetry codes: (i) 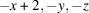 ; (iii) 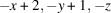 ; (v) 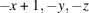 ; (vi) 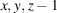 .

**Table 3:** Experimental details

Crystal data	
Chemical formula	[Ni(C_12_H_14_O_4_)(C_11_H_9_N_3_O)(H_2_O)]·H_2_O
*M*_r_	516.18
Crystal system, space group	Triclinic, *P* 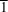
Temperature (K)	173
*a*, *b*, *c* (Å)	10.718 (4), 10.933 (3), 11.586 (3)
α, β, γ (°)	113.462 (4), 109.626 (4), 96.053 (4)
*V* (Å^3^)	1127.2 (6)
*Z*	2
Radiation type	Mo *K*α
μ (mm^−1^)	0.91
Crystal size (mm)	0.35 × 0.31 × 0.18

Data collection	
Diffractometer	Bruker APEXII CCD
No. of measured, independent and observed [*I* > 2σ(*I*)] reflections	12251, 4169, 3363
*R*_int_	0.037
(sin θ/λ)_max_ (Å^−1^)	0.606

Refinement	
*R*[*F* ^2^ > 2σ(*F* ^2^)], *wR*(*F* ^2^), *S*	0.042, 0.115, 1.07
No. of reflections	4169
No. of parameters	339
No. of restraints	7
H-atom treatment	H atoms treated by a mixture of independent and constrained refinement
Δρ_max_, Δρ_min_ (e Å^−3^)	0.82, −0.39

Computer programs: *COSMO* (Bruker, 2009 ▸), *APEX2* (Bruker, 2013 ▸), *SAINT* (Bruker, 2013 ▸), *olex2.solve* (Bourhis *et al.*, 2015 ▸), *SHELXTL* (Sheldrick, 2015 ▸), *CrystalMakerX* (Palmer, 2020 ▸), and *OLEX2* (Dolomanov *et al.*, 2009 ▸).

## full crystallographic data

### Crystal data

**Table:**

[Ni(C_12_H_14_O_4_)(C_11_H_9_N_3_O)(H_2_O)]·H_2_O	*Z* = 2
*M**_r_* = 516.18	*F*(000) = 540
Triclinic, *P*1	*D*_x_ = 1.521 Mg m^−^^3^
*a* = 10.718 (4) Å	Mo *K*α radiation, λ = 0.71073 Å
*b* = 10.933 (3) Å	Cell parameters from 6384 reflections
*c* = 11.586 (3) Å	θ = 2.2–25.4°
α = 113.462 (4)°	µ = 0.91 mm^−^^1^
β = 109.626 (4)°	*T* = 173 K
γ = 96.053 (4)°	Block, green
*V* = 1127.2 (6) Å^3^	0.35 × 0.31 × 0.18 mm

### Data collection

**Table:**

Bruker APEXII CCD diffractometer	3363 reflections with *I* > 2σ(*I*)
Radiation source: sealed tube	*R*_int_ = 0.037
Graphite monochromator	θ_max_ = 25.5°, θ_min_ = 2.1°
Detector resolution: 836.6 pixels mm^-1^	*h* = −12→12
φ and ω scans	*k* = −13→13
12251 measured reflections	*l* = −14→13
4169 independent reflections	

### Refinement

**Table:**

Refinement on *F*^2^	Primary atom site location: iterative
Least-squares matrix: full	Hydrogen site location: mixed
*R*[*F*^2^ > 2σ(*F*^2^)] = 0.042	H atoms treated by a mixture of independent and constrained refinement
*wR*(*F*^2^) = 0.115	*w* = 1/[σ^2^(*F*_o_^2^) + (0.062*P*)^2^ + 0.5423*P*] where *P* = (*F*_o_^2^ + 2*F*_c_^2^)/3
*S* = 1.07	(Δ/σ)_max_ < 0.001
4169 reflections	Δρ_max_ = 0.82 e Å^−^^3^
339 parameters	Δρ_min_ = −0.38 e Å^−^^3^
7 restraints	

### Special details

**Table:**

Geometry. All esds (except the esd in the dihedral angle between two l.s. planes) are estimated using the full covariance matrix. The cell esds are taken into account individually in the estimation of esds in distances, angles and torsion angles; correlations between esds in cell parameters are only used when they are defined by crystal symmetry. An approximate (isotropic) treatment of cell esds is used for estimating esds involving l.s. planes.

### Fractional atomic coordinates and isotropic or equivalent isotropic displacement parameters (Å^2^)

**Table:**

	*x*	*y*	*z*	*U*_iso_*/*U*_eq_	
Ni1	1.000000	0.000000	0.000000	0.01704 (15)	
Ni2	0.500000	0.500000	0.500000	0.01567 (15)	
O1	0.8747 (2)	0.02292 (19)	0.0998 (2)	0.0207 (4)	
O2	0.9879 (2)	0.2140 (2)	0.3019 (2)	0.0278 (5)	
O3	0.44548 (19)	0.28855 (18)	0.3693 (2)	0.0194 (4)	
O4	0.64885 (19)	0.39367 (18)	0.54244 (19)	0.0185 (4)	
O5	1.2822 (2)	0.3913 (2)	−0.0396 (2)	0.0257 (5)	
O6	0.8399 (2)	−0.1579 (2)	−0.1770 (2)	0.0323 (5)	
H6A	0.7521 (16)	−0.180 (3)	−0.228 (3)	0.049*	
H6B	0.880 (3)	−0.185 (4)	−0.231 (3)	0.049*	
N1	0.9443 (2)	0.1585 (2)	−0.0536 (2)	0.0196 (5)	
N2	1.4116 (2)	0.4695 (2)	−0.3776 (2)	0.0188 (5)	
N3	1.0701 (2)	0.3768 (2)	−0.1866 (2)	0.0205 (5)	
H3	1.027254	0.396693	−0.253463	0.043 (11)*	
C1	0.8886 (3)	0.1069 (3)	0.2187 (3)	0.0186 (6)	
C2	0.7255 (3)	0.1976 (3)	0.3250 (3)	0.0173 (6)	
H2A	0.684592	0.228008	0.254351	0.021*	
H2B	0.804113	0.275068	0.404075	0.013 (7)*	
C3	0.7765 (3)	0.0702 (3)	0.2634 (3)	0.0180 (6)	
C4	0.6536 (3)	−0.0489 (3)	0.1424 (3)	0.0214 (6)	
H4A	0.611146	−0.021060	0.069887	0.026*	
H4B	0.685396	−0.130982	0.101700	0.012 (7)*	
C5	0.8406 (3)	0.0254 (3)	0.3757 (3)	0.0231 (7)	
H5A	0.874709	−0.055787	0.337929	0.028*	
H5B	0.919763	0.102021	0.455123	0.017 (7)*	
C6	0.5469 (3)	−0.0859 (3)	0.1919 (3)	0.0253 (7)	
H6	0.467734	−0.164142	0.111628	0.021 (8)*	
C7	0.4942 (3)	0.0395 (3)	0.2508 (3)	0.0218 (6)	
H7A	0.423551	0.015057	0.281145	0.026*	
H7B	0.451510	0.067798	0.178771	0.011 (7)*	
C8	0.6825 (3)	0.1151 (3)	0.4850 (3)	0.0238 (7)	
H8A	0.761453	0.192252	0.564112	0.029*	
H8B	0.614538	0.092712	0.519184	0.033 (9)*	
C9	0.7322 (3)	−0.0122 (3)	0.4243 (3)	0.0274 (7)	
H9	0.774107	−0.041601	0.496624	0.030 (9)*	
C10	0.6096 (4)	−0.1307 (3)	0.3019 (4)	0.0316 (8)	
H10A	0.640577	−0.213959	0.263009	0.038*	
H10B	0.539579	−0.154594	0.333230	0.027 (8)*	
C11	0.6161 (3)	0.1596 (3)	0.3742 (3)	0.0175 (6)	
C12	0.5672 (3)	0.2864 (3)	0.4325 (3)	0.0170 (6)	
C14	0.8279 (3)	0.1933 (3)	−0.0554 (3)	0.0240 (7)	
H14	0.770731	0.149260	−0.027780	0.030 (9)*	
C15	1.0241 (3)	0.2222 (3)	−0.0922 (3)	0.0204 (6)	
H15	1.108039	0.199666	−0.089106	0.039 (10)*	
C16	0.7881 (3)	0.2911 (3)	−0.0962 (3)	0.0239 (7)	
H16	0.705149	0.313949	−0.095664	0.028 (8)*	
C17	0.9894 (3)	0.3197 (3)	−0.1365 (3)	0.0188 (6)	
C18	0.8696 (3)	0.3553 (3)	−0.1376 (3)	0.0220 (6)	
H18	0.843662	0.422547	−0.166249	0.022 (8)*	
C19	1.2793 (3)	0.3958 (3)	−0.4378 (3)	0.0204 (6)	
H19	1.233694	0.352711	−0.535775	0.002 (6)*	
C20	1.2063 (3)	0.3793 (3)	−0.3659 (3)	0.0204 (6)	
H20	1.112088	0.327124	−0.413126	0.016 (7)*	
C21	1.2716 (3)	0.4397 (3)	−0.2230 (3)	0.0173 (6)	
C22	1.4077 (3)	0.5186 (3)	−0.1589 (3)	0.0193 (6)	
H22	1.455078	0.562638	−0.061051	0.015 (7)*	
C23	1.4739 (3)	0.5327 (3)	−0.2388 (3)	0.0211 (6)	
H23	1.566465	0.588835	−0.194019	0.034 (9)*	
C24	1.2087 (3)	0.4033 (3)	−0.1390 (3)	0.0193 (6)	
O1W	0.9387 (2)	0.5047 (2)	−0.3366 (3)	0.0396 (6)	
H1WA	0.8521 (19)	0.483 (4)	−0.370 (4)	0.059*	
H1WB	0.969 (4)	0.590 (2)	−0.312 (4)	0.059*	

### Atomic displacement parameters (Å^2^)

**Table:**

	*U*^11^	*U*^22^	*U*^33^	*U*^12^	*U*^13^	*U*^23^
Ni1	0.0171 (3)	0.0182 (3)	0.0183 (3)	0.0030 (2)	0.0112 (2)	0.0079 (2)
Ni2	0.0158 (3)	0.0161 (3)	0.0184 (3)	0.0053 (2)	0.0106 (2)	0.0079 (2)
O1	0.0223 (11)	0.0220 (10)	0.0215 (11)	0.0055 (8)	0.0152 (9)	0.0086 (9)
O2	0.0206 (11)	0.0250 (11)	0.0283 (12)	−0.0013 (9)	0.0137 (10)	0.0024 (9)
O3	0.0157 (11)	0.0188 (9)	0.0242 (11)	0.0059 (8)	0.0093 (9)	0.0094 (9)
O4	0.0179 (11)	0.0182 (9)	0.0207 (11)	0.0059 (8)	0.0093 (9)	0.0088 (9)
O5	0.0213 (11)	0.0356 (12)	0.0254 (11)	0.0057 (9)	0.0089 (10)	0.0201 (10)
O6	0.0156 (11)	0.0406 (13)	0.0269 (13)	0.0016 (10)	0.0110 (10)	0.0024 (10)
N1	0.0183 (13)	0.0221 (12)	0.0204 (13)	0.0038 (10)	0.0103 (11)	0.0103 (10)
N2	0.0178 (13)	0.0207 (12)	0.0214 (13)	0.0054 (10)	0.0103 (11)	0.0111 (10)
N3	0.0198 (13)	0.0272 (12)	0.0234 (13)	0.0070 (10)	0.0108 (11)	0.0183 (11)
C1	0.0200 (16)	0.0212 (14)	0.0218 (15)	0.0103 (12)	0.0125 (13)	0.0125 (13)
C2	0.0170 (15)	0.0172 (13)	0.0210 (15)	0.0037 (11)	0.0122 (13)	0.0086 (12)
C3	0.0189 (15)	0.0208 (13)	0.0200 (15)	0.0066 (12)	0.0139 (13)	0.0095 (12)
C4	0.0186 (15)	0.0212 (14)	0.0249 (16)	0.0066 (12)	0.0135 (13)	0.0072 (13)
C5	0.0232 (17)	0.0285 (15)	0.0283 (17)	0.0144 (13)	0.0153 (14)	0.0176 (14)
C6	0.0229 (17)	0.0165 (14)	0.0337 (18)	0.0006 (12)	0.0186 (15)	0.0047 (13)
C7	0.0180 (15)	0.0176 (13)	0.0326 (17)	0.0039 (12)	0.0171 (14)	0.0092 (13)
C8	0.0305 (18)	0.0300 (15)	0.0277 (17)	0.0146 (14)	0.0215 (15)	0.0197 (14)
C9	0.0336 (19)	0.0363 (17)	0.0376 (19)	0.0212 (15)	0.0267 (16)	0.0280 (16)
C10	0.042 (2)	0.0187 (14)	0.052 (2)	0.0130 (14)	0.0340 (18)	0.0201 (15)
C11	0.0170 (15)	0.0180 (13)	0.0211 (15)	0.0065 (11)	0.0124 (13)	0.0084 (12)
C12	0.0187 (16)	0.0182 (13)	0.0186 (15)	0.0047 (12)	0.0121 (13)	0.0092 (12)
C14	0.0224 (16)	0.0247 (15)	0.0284 (17)	0.0039 (13)	0.0149 (14)	0.0124 (13)
C15	0.0186 (15)	0.0215 (14)	0.0227 (15)	0.0047 (12)	0.0107 (13)	0.0102 (12)
C16	0.0178 (16)	0.0277 (15)	0.0296 (17)	0.0075 (12)	0.0140 (14)	0.0127 (14)
C17	0.0182 (15)	0.0199 (13)	0.0198 (15)	0.0029 (11)	0.0106 (13)	0.0090 (12)
C18	0.0223 (16)	0.0222 (14)	0.0240 (16)	0.0052 (12)	0.0120 (13)	0.0114 (13)
C19	0.0223 (16)	0.0219 (14)	0.0161 (15)	0.0045 (12)	0.0078 (13)	0.0085 (12)
C20	0.0141 (15)	0.0241 (14)	0.0233 (16)	0.0028 (12)	0.0080 (13)	0.0114 (13)
C21	0.0195 (15)	0.0170 (13)	0.0223 (15)	0.0084 (11)	0.0130 (13)	0.0112 (12)
C22	0.0180 (15)	0.0226 (14)	0.0181 (15)	0.0039 (12)	0.0069 (13)	0.0109 (12)
C23	0.0179 (16)	0.0234 (14)	0.0234 (16)	0.0032 (12)	0.0096 (13)	0.0118 (13)
C24	0.0199 (16)	0.0190 (13)	0.0206 (15)	0.0055 (12)	0.0098 (13)	0.0092 (12)
O1W	0.0216 (12)	0.0367 (13)	0.0624 (18)	0.0055 (11)	0.0071 (13)	0.0337 (14)

### Geometric parameters (Å, º)

**Table:**

Ni1—O1^i^	2.0216 (19)	C5—H5B	0.9900
Ni1—O1	2.0216 (19)	C5—C9	1.536 (4)
Ni1—O6^i^	2.079 (2)	C6—H6	1.0000
Ni1—O6	2.079 (2)	C6—C7	1.531 (4)
Ni1—N1	2.138 (2)	C6—C10	1.519 (4)
Ni1—N1^i^	2.138 (2)	C7—H7A	0.9900
Ni2—O3	2.0766 (18)	C7—H7B	0.9900
Ni2—O3^ii^	2.0766 (18)	C7—C11	1.537 (4)
Ni2—O4	2.1113 (18)	C8—H8A	0.9900
Ni2—O4^ii^	2.1113 (18)	C8—H8B	0.9900
Ni2—N2^iii^	2.058 (2)	C8—C9	1.531 (4)
Ni2—N2^iv^	2.058 (2)	C8—C11	1.533 (4)
O1—C1	1.260 (3)	C9—H9	1.0000
O2—C1	1.258 (3)	C9—C10	1.530 (5)
O3—C12	1.272 (3)	C10—H10A	0.9900
O4—C12	1.269 (3)	C10—H10B	0.9900
O5—C24	1.224 (3)	C11—C12	1.515 (3)
O6—H6A	0.871 (14)	C14—H14	0.9500
O6—H6B	0.849 (18)	C14—C16	1.382 (4)
N1—C14	1.337 (4)	C15—H15	0.9500
N1—C15	1.340 (4)	C15—C17	1.389 (4)
N2—C19	1.339 (4)	C16—H16	0.9500
N2—C23	1.344 (4)	C16—C18	1.382 (4)
N3—H3	0.8800	C17—C18	1.378 (4)
N3—C17	1.410 (3)	C18—H18	0.9500
N3—C24	1.351 (4)	C19—H19	0.9500
C1—C3	1.534 (4)	C19—C20	1.366 (4)
C2—H2A	0.9900	C20—H20	0.9500
C2—H2B	0.9900	C20—C21	1.386 (4)
C2—C3	1.542 (4)	C21—C22	1.385 (4)
C2—C11	1.551 (4)	C21—C24	1.500 (4)
C3—C4	1.530 (4)	C22—H22	0.9500
C3—C5	1.544 (4)	C22—C23	1.384 (4)
C4—H4A	0.9900	C23—H23	0.9500
C4—H4B	0.9900	O1W—H1WA	0.840 (18)
C4—C6	1.526 (4)	O1W—H1WB	0.842 (18)
C5—H5A	0.9900		
			
O1^i^—Ni1—O1	180.0	C7—C6—H6	109.0
O1—Ni1—O6^i^	90.58 (8)	C10—C6—C4	110.4 (2)
O1—Ni1—O6	89.42 (8)	C10—C6—H6	109.0
O1^i^—Ni1—O6^i^	89.42 (8)	C10—C6—C7	109.6 (3)
O1^i^—Ni1—O6	90.58 (8)	C6—C7—H7A	109.9
O1—Ni1—N1	90.06 (8)	C6—C7—H7B	109.9
O1—Ni1—N1^i^	89.94 (8)	C6—C7—C11	108.9 (2)
O1^i^—Ni1—N1^i^	90.06 (8)	H7A—C7—H7B	108.3
O1^i^—Ni1—N1	89.94 (8)	C11—C7—H7A	109.9
O6^i^—Ni1—O6	180.0	C11—C7—H7B	109.9
O6—Ni1—N1	93.49 (9)	H8A—C8—H8B	108.2
O6^i^—Ni1—N1	86.51 (9)	C9—C8—H8A	109.7
O6^i^—Ni1—N1^i^	93.49 (9)	C9—C8—H8B	109.7
O6—Ni1—N1^i^	86.51 (9)	C9—C8—C11	109.9 (2)
N1—Ni1—N1^i^	180.0	C11—C8—H8A	109.7
O3^ii^—Ni2—O3	180.0	C11—C8—H8B	109.7
O3—Ni2—O4^ii^	116.95 (7)	C5—C9—H9	109.5
O3—Ni2—O4	63.05 (7)	C8—C9—C5	109.1 (2)
O3^ii^—Ni2—O4	116.95 (7)	C8—C9—H9	109.5
O3^ii^—Ni2—O4^ii^	63.05 (7)	C10—C9—C5	109.9 (3)
O4—Ni2—O4^ii^	180.0	C10—C9—C8	109.3 (3)
N2^iii^—Ni2—O3^ii^	92.34 (8)	C10—C9—H9	109.5
N2^iii^—Ni2—O3	87.67 (8)	C6—C10—C9	109.3 (2)
N2^iv^—Ni2—O3	92.33 (8)	C6—C10—H10A	109.8
N2^iv^—Ni2—O3^ii^	87.66 (8)	C6—C10—H10B	109.8
N2^iv^—Ni2—O4	94.86 (8)	C9—C10—H10A	109.8
N2^iii^—Ni2—O4	85.14 (8)	C9—C10—H10B	109.8
N2^iii^—Ni2—O4^ii^	94.86 (8)	H10A—C10—H10B	108.3
N2^iv^—Ni2—O4^ii^	85.14 (8)	C7—C11—C2	109.4 (2)
N2^iii^—Ni2—N2^iv^	180.0	C8—C11—C2	108.7 (2)
C1—O1—Ni1	133.44 (18)	C8—C11—C7	109.7 (2)
C12—O3—Ni2	89.28 (15)	C12—C11—C2	108.2 (2)
C12—O4—Ni2	87.83 (15)	C12—C11—C7	110.0 (2)
Ni1—O6—H6A	143 (2)	C12—C11—C8	110.8 (2)
Ni1—O6—H6B	101 (2)	O3—C12—Ni2	59.03 (13)
H6A—O6—H6B	107 (3)	O3—C12—C11	120.5 (2)
C14—N1—Ni1	121.94 (19)	O4—C12—Ni2	60.60 (13)
C14—N1—C15	118.1 (2)	O4—C12—O3	119.1 (2)
C15—N1—Ni1	119.93 (18)	O4—C12—C11	120.4 (2)
C19—N2—Ni2^v^	118.35 (19)	C11—C12—Ni2	170.38 (19)
C19—N2—C23	117.7 (2)	N1—C14—H14	118.9
C23—N2—Ni2^v^	123.49 (19)	N1—C14—C16	122.2 (3)
C17—N3—H3	117.7	C16—C14—H14	118.9
C24—N3—H3	117.7	N1—C15—H15	118.6
C24—N3—C17	124.6 (2)	N1—C15—C17	122.8 (3)
O1—C1—C3	116.0 (2)	C17—C15—H15	118.6
O2—C1—O1	124.8 (3)	C14—C16—H16	120.2
O2—C1—C3	119.1 (2)	C18—C16—C14	119.6 (3)
H2A—C2—H2B	108.2	C18—C16—H16	120.2
C3—C2—H2A	109.7	C15—C17—N3	121.4 (2)
C3—C2—H2B	109.7	C18—C17—N3	119.7 (2)
C3—C2—C11	109.7 (2)	C18—C17—C15	118.8 (3)
C11—C2—H2A	109.7	C16—C18—H18	120.8
C11—C2—H2B	109.7	C17—C18—C16	118.5 (3)
C1—C3—C2	110.6 (2)	C17—C18—H18	120.8
C1—C3—C5	107.9 (2)	N2—C19—H19	118.2
C2—C3—C5	108.7 (2)	N2—C19—C20	123.5 (3)
C4—C3—C1	111.7 (2)	C20—C19—H19	118.2
C4—C3—C2	109.0 (2)	C19—C20—H20	120.5
C4—C3—C5	108.9 (2)	C19—C20—C21	119.0 (3)
C3—C4—H4A	109.6	C21—C20—H20	120.5
C3—C4—H4B	109.6	C20—C21—C24	122.1 (2)
H4A—C4—H4B	108.1	C22—C21—C20	118.1 (3)
C6—C4—C3	110.1 (2)	C22—C21—C24	119.0 (2)
C6—C4—H4A	109.6	C21—C22—H22	120.3
C6—C4—H4B	109.6	C23—C22—C21	119.4 (3)
C3—C5—H5A	109.7	C23—C22—H22	120.3
C3—C5—H5B	109.7	N2—C23—C22	122.1 (3)
H5A—C5—H5B	108.2	N2—C23—H23	118.9
C9—C5—C3	109.9 (2)	C22—C23—H23	118.9
C9—C5—H5A	109.7	O5—C24—N3	124.6 (3)
C9—C5—H5B	109.7	O5—C24—C21	119.4 (3)
C4—C6—H6	109.0	N3—C24—C21	115.8 (2)
C4—C6—C7	109.8 (2)	H1WA—O1W—H1WB	109 (3)
			
Ni1—O1—C1—O2	9.4 (4)	C5—C9—C10—C6	−59.2 (3)
Ni1—O1—C1—C3	−168.59 (17)	C6—C7—C11—C2	59.9 (3)
Ni1—N1—C14—C16	−176.9 (2)	C6—C7—C11—C8	−59.2 (3)
Ni1—N1—C15—C17	175.9 (2)	C6—C7—C11—C12	178.7 (2)
Ni2—O3—C12—O4	8.7 (2)	C7—C6—C10—C9	−61.6 (3)
Ni2—O3—C12—C11	−168.8 (2)	C7—C11—C12—O3	−8.7 (3)
Ni2—O4—C12—O3	−8.5 (2)	C7—C11—C12—O4	173.8 (2)
Ni2—O4—C12—C11	169.0 (2)	C8—C9—C10—C6	60.4 (3)
Ni2^v^—N2—C19—C20	−173.9 (2)	C8—C11—C12—O3	−130.2 (3)
Ni2^v^—N2—C23—C22	174.8 (2)	C8—C11—C12—O4	52.3 (3)
O1—C1—C3—C2	−132.5 (2)	C9—C8—C11—C2	−60.7 (3)
O1—C1—C3—C4	−10.9 (3)	C9—C8—C11—C7	58.8 (3)
O1—C1—C3—C5	108.7 (3)	C9—C8—C11—C12	−179.5 (2)
O2—C1—C3—C2	49.3 (3)	C10—C6—C7—C11	60.8 (3)
O2—C1—C3—C4	170.9 (2)	C11—C2—C3—C1	−178.0 (2)
O2—C1—C3—C5	−69.5 (3)	C11—C2—C3—C4	58.8 (3)
N1—C14—C16—C18	0.4 (4)	C11—C2—C3—C5	−59.7 (3)
N1—C15—C17—N3	−175.0 (2)	C11—C8—C9—C5	61.0 (3)
N1—C15—C17—C18	1.7 (4)	C11—C8—C9—C10	−59.2 (3)
N2—C19—C20—C21	−0.9 (4)	C14—N1—C15—C17	−1.5 (4)
N3—C17—C18—C16	175.9 (3)	C14—C16—C18—C17	−0.2 (4)
C1—C3—C4—C6	177.9 (2)	C15—N1—C14—C16	0.4 (4)
C1—C3—C5—C9	179.9 (2)	C15—C17—C18—C16	−0.8 (4)
C2—C3—C4—C6	−59.6 (3)	C17—N3—C24—O5	−6.1 (4)
C2—C3—C5—C9	59.9 (3)	C17—N3—C24—C21	169.4 (2)
C2—C11—C12—O3	110.7 (3)	C19—N2—C23—C22	2.8 (4)
C2—C11—C12—O4	−66.8 (3)	C19—C20—C21—C22	2.2 (4)
C3—C2—C11—C7	−59.5 (3)	C19—C20—C21—C24	−167.4 (2)
C3—C2—C11—C8	60.3 (3)	C20—C21—C22—C23	−1.0 (4)
C3—C2—C11—C12	−179.3 (2)	C20—C21—C24—O5	140.1 (3)
C3—C4—C6—C7	61.0 (3)	C20—C21—C24—N3	−35.7 (4)
C3—C4—C6—C10	−60.0 (3)	C21—C22—C23—N2	−1.6 (4)
C3—C5—C9—C8	−60.5 (3)	C22—C21—C24—O5	−29.4 (4)
C3—C5—C9—C10	59.4 (3)	C22—C21—C24—N3	154.9 (2)
C4—C3—C5—C9	−58.8 (3)	C23—N2—C19—C20	−1.6 (4)
C4—C6—C7—C11	−60.8 (3)	C24—N3—C17—C15	−34.5 (4)
C4—C6—C10—C9	59.5 (3)	C24—N3—C17—C18	148.9 (3)
C5—C3—C4—C6	58.9 (3)	C24—C21—C22—C23	168.9 (2)

Symmetry codes: (i) −*x*+2, −*y*, −*z*; (ii) −*x*+1, −*y*+1, −*z*+1; (iii) −*x*+2, −*y*+1, −*z*; (iv) *x*−1, *y*, *z*+1; (v) *x*+1, *y*, *z*−1.

### Hydrogen-bond geometry (Å, º)

**Table:**

*D*—H···*A*	*D*—H	H···*A*	*D*···*A*	*D*—H···*A*
O6—H6*A*···O3^vi^	0.87 (1)	2.04 (2)	2.886 (3)	163 (3)
O6—H6*B*···O2^i^	0.85 (2)	1.86 (2)	2.684 (3)	163 (3)
N3—H3···O1*W*	0.88	1.94	2.769 (3)	157
O1*W*—H1*WA*···O4^vii^	0.84 (2)	2.00 (2)	2.831 (3)	169 (4)
O1*W*—H1*WB*···O2^iii^	0.84 (2)	2.10 (2)	2.921 (3)	165 (4)

Symmetry codes: (i) −*x*+2, −*y*, −*z*; (iii) −*x*+2, −*y*+1, −*z*; (vi) −*x*+1, −*y*, −*z*; (vii) *x*, *y*, *z*−1.
